# Trends in analgesia in prehospital trauma care: an analysis of 105.908 patients from the multicenter database TraumaRegister DGU^®^

**DOI:** 10.1186/s12873-025-01186-z

**Published:** 2025-03-05

**Authors:** Davut Deniz Uzun, Jan-Philipp Stock, Richard Steffen, Jürgen Knapp, Rolf Lefering, Felix C. F. Schmitt, Markus A. Weigand, Matthias Münzberg, Christoph G. Woelfl, David Häske

**Affiliations:** 1 Medical Faculty Heidelberg, Department of Anesthesiology, Heidelberg University, Heidelberg, Germany; 2 Department of Anesthesiology, Intensive Care Medicine, Emergency and Pain Medicine, Klinikum am Steinenberg, Reutlingen, Germany; 3 Department of Anaesthesiology and Pain Medicine, Bern University Hospital, Inselspital, University of Bern, Bern, Switzerland; 4 Institute for Research in Operative Medicine (IFOM), University Witten/Herdecke, Cologne, Germany; 5 Department of Trauma and Orthopedic Surgery BG Trauma Center Frankfurt, Frankfurt am Main, Germany; 6 Department of Orthopaedics and Trauma Surgery, Marienhaus Hospital Neuwied, Neuwied, Germany; 7 Center of Public Health and Health Services Research, University Hospital Tübingen, Tübingen, Germany; 8 Emergency Medical Service, German Red Cross, Reutlingen, Germany

**Keywords:** Analgesia, Trauma, Pain, Severely injured patients, Trauma register

## Abstract

**Background:**

The management of pain in patients with traumatic injuries is a common task for emergency medicine providers, particularly in the prehospital setting. However, for sufficient and safe analgesia, correct pain recording and documentation is also necessary. The aim of this study was to assess trends in analgesia over the study period and to identify factors that may enable more sufficient pain management in trauma care.

**Methods:**

The TraumaRegister DGU^®^ recorded data of patients who were primarily treated at one of the participating hospitals between 2011 and 2020 and received analgesia as part of their prehospital care. This retrospective analysis included a total of 105.908 severely injured patients from Germany, Switzerland, and Austria. Patients with and without analgesia were compared, and factors associated with analgesia were investigated with logistic regression analysis.

**Results:**

The mean age of the patients enrolled was 50 ± 22 years. 71% were male and 29% were female. Out of all the patients, 66% (*n* = 70,257) received prehospital analgesia. The average age of patients in the analgesia group was 48 ± 21 years, the non-analgesia group had an average age of 54 ± 23 years. 67% of the male patients received analgesia compared to 64% of the female patients. The mean Injury Severity Score (ISS) in the analgesia group was 21.2 points, compared to 16.5 points in the non-analgesia group. 4% of the patients were under the age of sixteen, and of these, 65% received analgesia. 29% of patients were older than 65 years and received analgesia in 57%. Presence of an emergency physician at scene, was a remarkable independent variable for the receipt of analgesia (Odds Ratio 5.55; *p* < 0.001). Transportation by helicopter was also a significant predictor for analgesia (OR 1.62; *p* < 0.001).

**Conclusions:**

Analgesia is a crucial aspect of emergency medicine, as evidenced by relevant guidelines. Nevertheless, it is plausible that a considerable proportion of seriously injured patients do not receive optimal analgesic treatment, or at the very least, this is not documented. In this regard, both aspects require optimization.

## Background

Pain is a common, highly variable, and subjective symptom in injured emergency patients [1]. In Germany, approximately 18,000 patients with severe injuries are treated by the emergency medical services (EMS) each year [2]. Patients with both multiple and single injuries require appropriate analgesia and, in some cases, additional sedation. This is a central pillar of emergency medical care at the accident site and emergency department [3–5].

In the history of trauma care, the focus has been on identifying and treating potentially lethal injuries first, according to the principle of “treat what kills first”. However, it is increasingly recognised that adequate and early analgesia is also crucial, if possible simultaneously with the treatment of life-threatening injuries [6, 7]. The primary goal of analgesia should be to reduce pain and stress during the acute phase while also preventing physiological pain reactions and post-traumatic responses [7]. Acute pain can activate the autonomic nervous system, causing a sympathoadrenergic stress response. This response may cause symptoms such as tachycardia, sweating, increased respiratory rate, and hypertension. These symptoms can increase myocardial oxygen consumption, which is especially concerning for patients with pre-existing coronary artery disease [7]. Therefore, the aim is to improve the patient’s well-being by reducing stress and pain through adequate analgesia and to prevent the negative effects of pain and the resulting potential health or vital threats [8, 9].

Research has shown that various factors, including patient and practitioner characteristics, can affect the effectiveness of pain relief. For example, studies have found that the experience level and gender of emergency practitioners may impact pain management outcomes [6, 10–12]. Additionally, since the administration of analgesia can depend on the provider, there may be limitations in paramedic emergency services where an emergency physician is not present [13].

While pain management is a fundamental requirement for healthcare professionals, patients often receive inadequate pain management, as current data shows [6, 10, 14–16]. This problem is commonly called ‘oligoanalgesia’ in the literature [15].

However, the topic of analgesia is not included in all common training programs, such as International Trauma Life Support (ITLS) and Prehospital Trauma Life Support (PHTLS), and as a result, it is not comprehensively addressed or systematically trained [17]. Only the latest version of the German evidence- and consensus-based guideline on the treatment of patients with severe/multiple injuries has added a separate chapter on analgesia, emphasizing its importance for patients, doctors, paramedics, and nursing staff. The chapter provides corresponding scientific recommendations [18].

However, the challenge lies not only in adequate training but also in the correct recording of pain. Successful pain management is a complex process in which various factors are involved. Nevertheless, if the guidelines already take analgesia into account, the documentation must be correct and thus enable an analysis of analgesia processes, safety and patient effect. Although the data from the TraumaRegister DGU^®^ (TR-DGU) does not permit a detailed discussion of pain, the frequency of analgesia in severely injured patients can be quantified.

This study aims to assess the trends and influences on analgesia in prehospital major trauma care, as recorded in the TraumaRegister DGU^®^ (TR-DGU).

## Methods

### Study design

A retrospective, multicenter, cross-sectional study was conducted based on routinely collected observational data from the TraumaRegister DGU^®^. The present study follows the publication guideline of the TR-DGU and is registered as TR-DGU project TR-DGU- 2021-031.

### Database

The TraumaRegister DGU^®^ (TR-DGU) of the German Trauma Society (DGU) was established in 1993. The aim of this multi-centre database is the pseudonymised and standardised documentation of severely injured patients.

Data are collected prospectively in four successive phases, starting at the scene of the accident and ending when discharged from hospital: (A) prehospital phase, (B) emergency room and initial operation, (C) intensive care unit, and (D) discharge.

Documentation includes detailed information on demographics, injury patterns, co-morbidities, pre- and in-hospital management, intensive care unit course and relevant laboratory findings (including transfusion data and results). The inclusion criterion is admission to the hospital via the emergency room with subsequent ICU/IMC care or arrival at the hospital with vital signs and death before admission to the ICU.

The infrastructure for documentation, data management, and data analysis is provided by the AUC-Akademie der Unfallchirurgie GmbH, a subsidiary of the German Trauma Society. The Committee on Emergency Medicine, Intensive Care and Trauma Management (Sektion NIS) of the German Trauma Society assumes the role of scientific leadership. The pseudonymised data is entered into a central database by the participating hospitals via a web-based application. Before publication, the scientific evaluation of the data is confirmed by a peer review process set up by the NIS Section. The participating hospitals are predominantly located in Germany (90%), but an increasing number of hospitals from other countries also contribute data (including Austria, Belgium, China, Finland, Luxembourg, Slovenia, Switzerland, the Netherlands, and the United Arab Emirates). Currently, more than 38,000 cases from almost 700 hospitals are entered into the database each year. Participation in the TraumaRegister DGU^®^ is voluntary. The TraumaNetzwerk DGU^®^ is a network of hospitals that have been organised according to uniform standards of care and quality assurance. They have been categorised into trauma centers of levels 1 to 3 based on their structure, personnel, and equipment resources, as well as their responsibilities, as outlined in the White Book on Trauma Care by the DGU^®^ [8]. Level 1 trauma centers represent centers with the highest resources, while level 3 centers have limited resources. However, for hospitals affiliated with the TraumaNetzwerk DGU^®^, the submission of at least a basic dataset is mandatory for quality assurance purposes.

This study was conducted in accordance with applicable laws and guidelines. Data collection is based on the nationwide obligation for quality assurance in trauma care and is the responsibility of the participating hospitals. This study was reviewed by the Review Board of the Trauma Registry DGU^®^. The study complies with the publication guidelines of the TraumaRegister DGU^®^ and is registered under the TR-DGU project ID 2021-031. Due to the retrospective study design and the anonymized data, no further ethics committee approval was required. The study was conducted in accordance with the most recent version of the Declaration of Helsinki.

### Classifications

Injury severity assessment the Abbreviated Injury Scale (AIS) is an anatomical coding system for classifying and describing the severity of injuries in every body region, and it is used in many trauma registries. Based on the AIS severity values, the injury severity score (ISS) can be calculated to assess the cumulative trauma severity. Severe injury is often defined by ISS ≥ 16.

### Patient selection

**Fig. 1:**
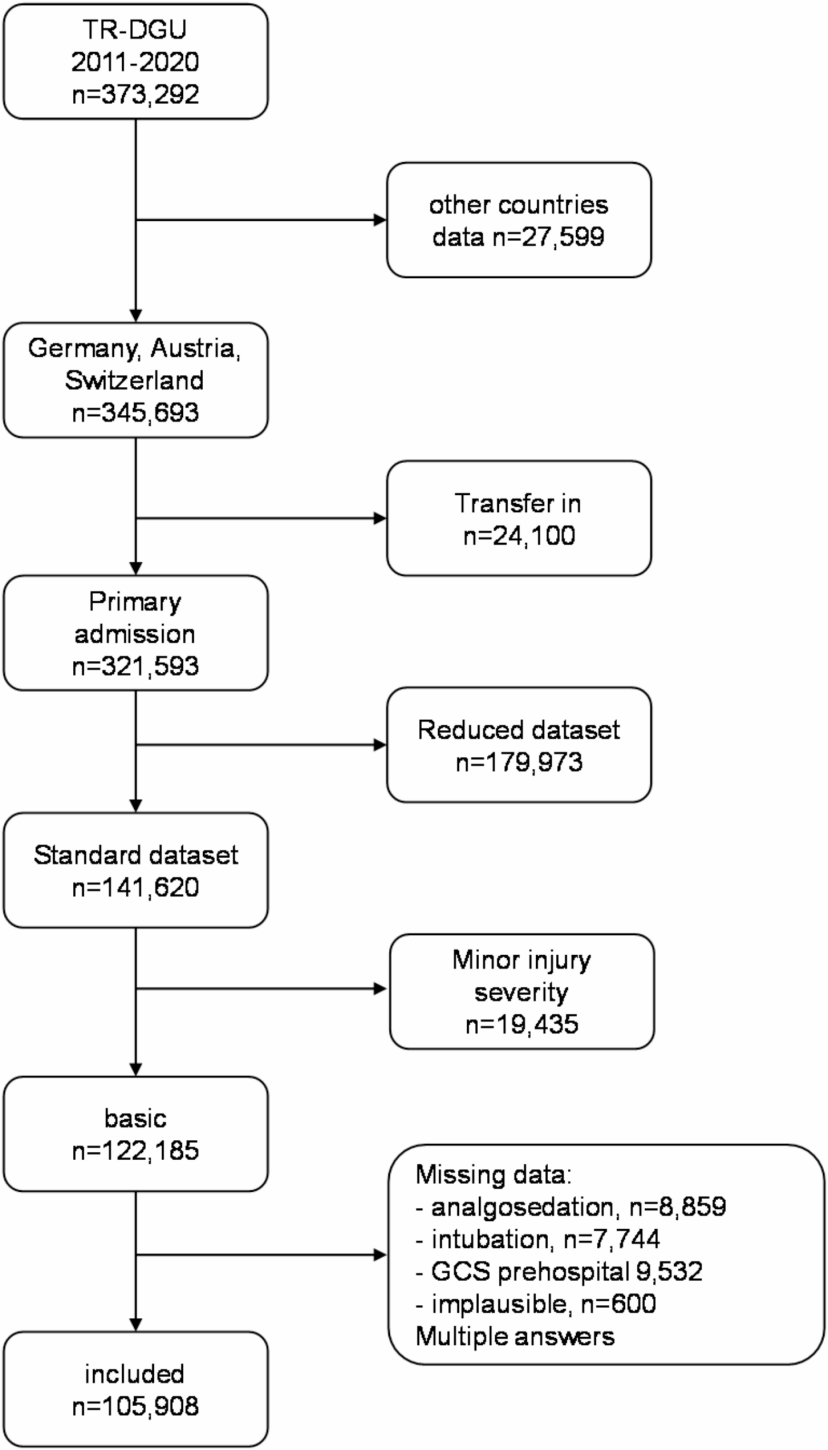
Patient selection

The study included patients who received primary treatment in a German, Austrian, or Swiss hospital from 2011 to 2020. We used the standard data set for this analysis, excluded patients with minor injuries and data sets with missing specific characteristics (Fig. 1). When evaluating analgesia, we focus on the variable “analgosedation”, continuously referred to as “analgesia”.

### Statistical analysis

For continuous variables, the data is presented as a mean with standard deviation (SD), or as median with quartiles in case of skewed data; categorical variables are presented as numbers with percentages. P-values < 0.05 were considered statistically significant. For the descriptive analysis, no p-values were given since the huge sample size would result in ‘significant’ results even in minor differences. A logistic regression analysis was conducted to identify predictors for applying analgesia (dependent variable). Regression coefficients are given with standard error and the respective p-value of the model, as well as odds ratios (OR) with a 95% confidence intervals (CI). Statistical analysis was performed using SPSS Version 28.0 (IBM Inc., Armonk, NY, USA).

## Results

### Baseline and demographic data

During the study period from 2011 to 2020, 105,908 patients (Fig. 1) from Germany, Switzerland, and Austria, were included. 71% were male and 29% female. The majority of the patients were from Germany (88%, *n* = 93,074), followed by Austria (8%, *n* = 8,121), and Switzerland (4%, *n* = 4,713). Overall, 66% (*n* = 70,238) of the patients received prehospital analgesia, of which 28% (*n* = 19,726) were female and 72% (*n* = 50,512) were male. Details on the different countries are shown in Table 1. In this evaluation, 64% of women received analgesia compared to 67% of men. The mean age of the patients in the analgesia group was 48 ± 22 years, while those from the non-analgesia group had a mean age of 54 ± 23 years. The mean Injury Severity Score (ISS) of the patients in the analgesia group was 21 ± 13, compared to 16 ± 10 for those from the non-analgesia group. 4.0% of the patients were under sixteen years, and 65% received prehospital analgesia.

29% (*n* = 30,988) of patients were older than 65 years. In this patient group older than 65 years, 57% (*n* = 17,619) of these patients received prehospital analgesia. Shock (systolic BP < 90 mmHg) was observed in 10% (*n* = 9,784) of the registered trauma patients. Of these patients, 76% (*n* = 7,464) received prehospital analgesia. Baseline patient data and parameters are shown in (Table 1).

**Table 1:** Baseline characteristics

	Analgesia	No analgesia
ISS, mean ± SD	21.2 ± 13.1	16.5 ± 10.9
BP systolic < 90 mmHg, n (%)	7,464 (76%)	2,320 (24%)
GCS ≤ 8, n (%)	17,750 (81%)	4,094 (19%)
Prehospital Intubation, n (%)	27,948 (93%)	2,183 (7%)
Fluid administration, n (%)	63,824 (70%)	27,232 (30%)
Catecholamine therapy n (%)	8,332 (87%)	1,229 (13%)
Chest Tube n (%)	2,938 (91%)	308 (9%)
On scene time, mean (min) ± SD	30.9 ± 17.0	23.1 ± 12.8
Number of prehospital interventions, ± SD	2.5 ± 0.9	0.9 ± 0.7
Germany	60,618 (65%)	32,456 (35%)
Austria	6,339 (78%)	1,782 (22%)
Switzerland	3,300 (70%)	1,413 (30%)

**Fig. 2:**
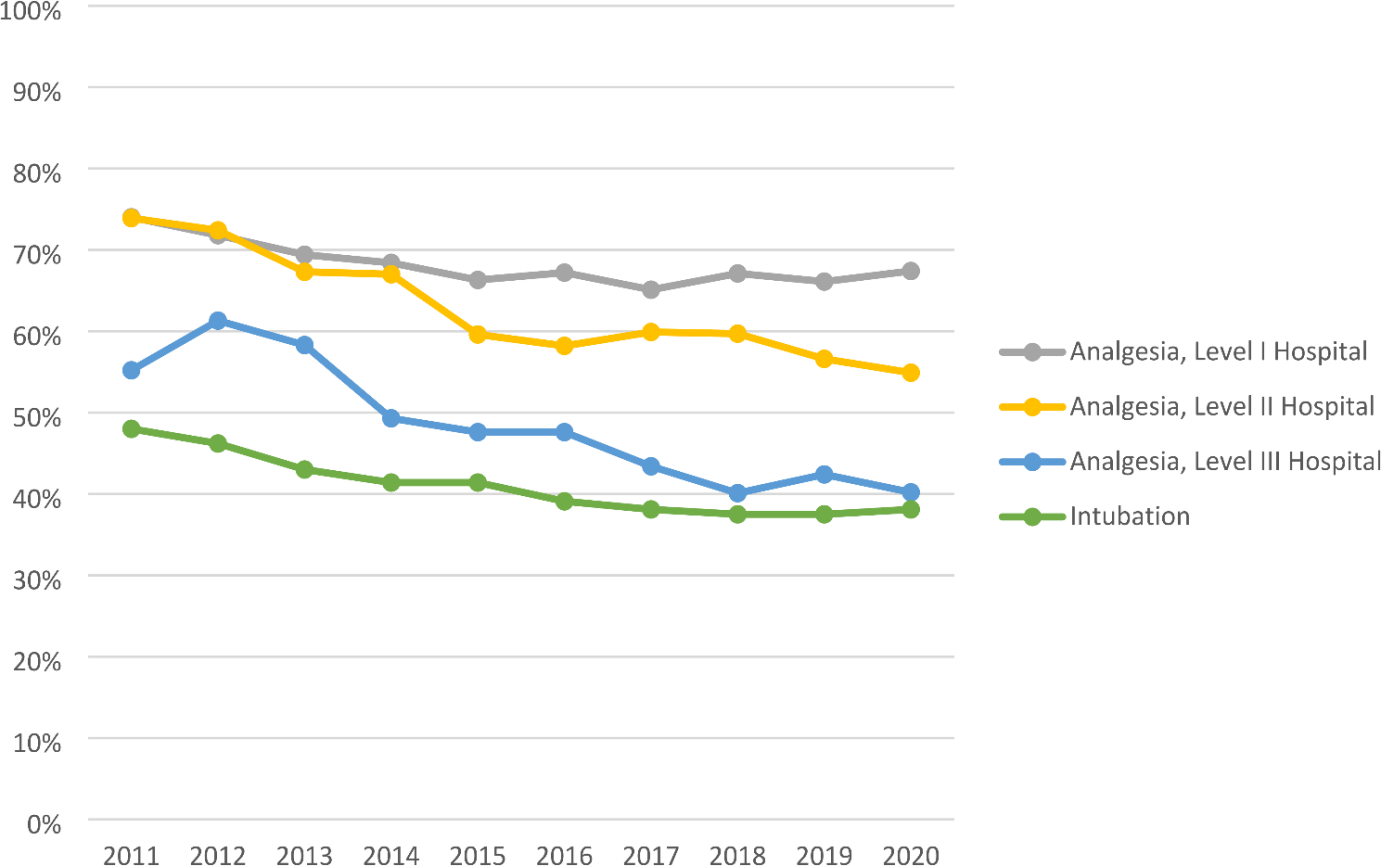
Frequency of prehospital analgesia, by hospital level of care, and endotracheal intubation during the study period (2011–2020)

The time course shows decreased prehospital analgesia frequency from 2011 to 2020. The visual progression is shown in (Fig. 2).

From 2011 to 2020, the frequency of analgesia decreased by 9% for patients admitted to Level I hospitals, by 26% for Level II hospitals, and by 27% for Level III hospitals. For comparison, advanced airway management (endotracheal intubation) decreased by 21% over the years.

### Cause of accident

51% (*n* = 54,265) had a traffic accident by car, motorbike, bicycle, or pedestrian. The details of the causes of accidents are set out in Table 2:

**Table 2:** The details of the causes of accidents

Cause of Accident	Collective total % (*n*)	Patients with analgesia %
Car	20% (*n* = 21,537)	73%
Motorbike	13% (*n* = 13,604)	78%
Bicycle	10% (*n* = 10,214)	59%
Pedestrian	7% (*n* = 7,080)	69%
High Fall (> 3 m)	17% (*n* = 17,304)	72%
Low Fall (< 3 m)	22% (*n* = 22,546)	51%
Other	11% (*n* = 12,264)	67%

### Type of trauma

In the current study, the majority of the patients 95% (*n* = 100,612), had blunt trauma as their mechanism of injury. Only 5% (*n* = 5,296) of the patients had a penetrating trauma. In the blunt trauma group, 67% (*n* = 67,410) of the patients received analgesia. In the patient group with penetrating trauma, 66% (*n* = 3,495) were in the analgesia group.

### Vitals

At the accident site, 21% (*n* = 21,844) of the population had an initial GCS score of 8 or less. Of these patients, 81% (*n* = 17,750) received analgesia. In the analgesia group, 76% had hemodynamic instability with shock (systolic blood pressure ≤ 90 mmHg) compared to 24% in the non-analgesic group.

### Structure analysis

Most of the patients in this analysis were treated during the day 62% (*n* = 66,064).

38% (*n* = 39,844) of the patients were treated at night. Of the patients treated during the day, 67% (*n* = 44,398) were in the analgesia group. In the patients treated during the night, 65% (*n* = 25,859) received prehospital analgesia.

55% (*n* = 58,722) of the patients were treated during the week (Monday-Thursday). Of these patients, 66% (*n* = 38,880) received analgesia during their prehospital treatment.

The remaining 45% (*n* = 47,186) of the patients were treated at the weekend (Friday to Sunday), of which 67% (*n* = 31,377) received prehospital analgesia.

Patients in the analgesia group had an on-scene time of 31 ± 16.95 min, compared to 23 ± 12.76 min for patients in the non-analgesia group. Of these trauma patients who received analgesia, 83% (*n* = 58,298) were transferred to a Level I trauma center, while 14% (*n* = 10,118) were transferred to a Level II trauma center. Only 3% (*n* = 1,841) of patients were transferred to a Level III trauma center.

### Presence of an emergency physician

The majority of patients were treated in the presence of an emergency physician, (92%, *n* = 95,497). In the presence of an emergency physician, 70.6% of patients received analgesia, compared with 20.4% of patients who received analgesia without an emergency physician.

### Ground transport vs. HEMS transport

In this analysis, 70% (*n* = 72,304) of the patients were transported to the trauma center by ground-based EMS, while 30% (*n* = 31,459) arrived by helicopter emergency medical services (HEMS). Of those transported by ground, 60% (*n* = 43,163) received prehospital analgesia, compared to 82% (*n* = 25,876) of those transported by HEMS.

### Prehospital procedures

During prehospital trauma care, 29% (*n* = 30,131) of the total population needed tracheal intubation for advanced airway management. According to the registry analysis, 92% (*n* = 27,948) of these patients received analgesia. However, 7% (*n* = 2,183) of patients did not receive analgesia despite being intubated.

3% (*n* = 3,246) of the trauma patients needed chest decompression (using a chest tube) in the prehospital setting. Of these patients, 91% (*n* = 2,938) also received analgesia. Of those patients, 95% (*n* = 63,824) received concomitant fluid administration. On average, patients in the analgesia group received 872 ml of fluid volume compared to patients in the non-analgesia group, who received 528 ml.

9% (*n* = 9,561) of the patients in the registry received prehospital catecholamines. 87% required catecholamines in the analgesia group, compared to 13% in the non-analgesia group.

**Table 3:** Multivariate logistic regression analysis to predict analgesia, *n* = 103,734 patients. SE = standard error, OR = odds ratio, CI = confidence interval, ref: reference category

Predictor	Coefficient	SE	OR	95% CI	*p*-Value
Age group (ref: <60 years)					
60–69 years	-0.225	0.024	0.79	0.76-0,83	< 0.001
70–79 years	-0.366	0.024	0.69	0.66–0.72	< 0.001
>80 years	-0.432	0.027	0.65	0.61–0.68	0.684
Male patient	-0.027	0.017	0.97	0.94–1.00	0.119
Relevant injuries (AIS ≥ 3)					
Head Chest Abdomen Extremities	-0.422 0.397 0.235 0.883	0.019 0.017 0.028 0.021	0.65 1.48 1.26 2.43	0.63–0.68 1.44–1.53 1.19–1.33 2.33–2.53	< 0.001 < 0.001 < 0.001 < 0.001
Physician at scene	1.713	0.035	5.54	5.18–5.93	< 0.001
HEMS transport	0.482	0.019	1.62	1.56–1.68	< 0.001
Country (ref.: Germany)					
Austria	0.569	0.033	1.76	1.65–1.88	< 0.001
Switzerland	1.042	0.043	2.83	2.60–3.08	< 0.001
GCS ≤ 8	-0.989	0.038	0.37	0.34–0.40	< 0.001
Shock (sPB ≤ 90mmHg)	-0.198	0.027	0.82	0.77–0.86	< 0.001
Low fall (< 3 m)	-0.264	0.020	0.76	0.73–0.79	< 0.001
Intubation prehospital	3.221	0.042	25.04	23.08–27.17	< 0.001
Chest decompression	-0.191	0.072	0.82	0.71–0.95	0.008
Fluid administration	0.857	0.023	2.35	2.25–2.46	< 0.001
Catecholamines	-0.351	0.043	0.70	0.64–0.76	< 0.001

Multivariate logistic regression analysis included *n* = 103,734 patients. The highest predictor for prehospital analgesia was prehospital intubation with OR 25.04, *p* < 0.001, followed by a physician on scene with OR = 5.55, *p* < 0.001. Austria (OR 1.77) and Switzerland (OR 2.83) applied analgesia much more frequently as Germans. Further predictors were extremity trauma OR 2.43 and volume infusion OR 2.36.

Unconsciousness (OR 0.37), head injury (OR 0.66) and the older patient groups were negatively associated (Table 3). The gender of the patient did not play a significant role.

## Discussion

Analgesia is one of the most essential measures in modern emergency medicine and can be analyzed for seriously injured patients using routine data in the TraumaRegister DGU^®^. The fact that only 66% of trauma patients received analgesia must be critically questioned.

In this study, only the quantity of analgesia was surveyed, regardless of the qualitative quality of analgesia and the subjective effect on the patients, as the patient’s pain was not documented in the TraumaRegister DGU^®^. It is, therefore, unknown how much pain the patients in the TraumaRegister DGU^®^ experienced and whether analgesia was desired. In addition, there appear to be hardly any public registers with insight into analgesia. While different target parameters are sometimes cited as successful analgesia, the German polytrauma guideline states that pain should be reduced to the Numeric Rating Scale ≤ 4 [19]. Other studies focus on quality and not just quantity. Helm et al. from a physician-staffed helicopter emergency medical service (p-HEMS) using the example of > 100,000 with GCS ≥ 8 that prehospital analgesia occurred in 82.9% of patients [15] although other experiences are around 60% [6, 10].

On the other hand, a study conducted on approximately 20,000 patients between the ages of 0 and 16 in the Air Ambulance, 58.8% of whom had trauma, reported successful analgesia in 95.1% of patients [20]. An analysis of all emergency physician interventions in a German federal state based on 50,526 patients showed successful analgesia in 93.3% [21].

This shows a not inconsiderable range of prehospital analgesia and indicates that oligo-analgesia also occurs in excellently trained systems. Inadequate analgesia can have several causes. One factor that is often underestimated in analyses of pain management prior to hospitalization is differences in the medical training and specialty of the physician. However, the gender and professional experience of the practitioner, the severity of the injury/illness, time and procedural aspects in patient care, and the basic influencing factors in pain assessment before analgesia are applied [21].

Figure 1 shows decreased analgesia over the years, particularly in level II and III hospitals. For instance 2011, 74% of patients in a level II hospital received analgesia, while in 2020, this figure decreased to 55%. Although the registry data has limitations, it is still unclear why analgesia has decreased despite the increasing professionalization of emergency medical services. The guidelines now recommend a more cautious approach to induction during prehospital intubation, but this does not extend to analgesia. It is unclear why there were differences in the frequency of prehospital analgesia depending on the level of care at the receiving hospitals. One possible explanation is that the emergency physicians typically attend their own hospital, and their training differs in relevant ways, thus influencing the quality of prehospital care and analgesia. However, this explanation may seem contrived to the authors.

This lack of clarity may also be due to the entry into the TraumaRegister DGU^®^ or the naming of the variable. The term ‘Analgosedierung’ (analgosedation) in German refers to a combination of analgesia and sedation. It is important to note that this does not refer to sedation in the sense of anesthesia and possibly consecutive intubation. However, the absence of an option to document anesthesia in the TraumaRegister DGU^®^ may lead to its inclusion under analgosedation. On the one hand, this would explain why 7% of intubated patients did not receive analgesia (because they did not receive anesthesia). One possible explanation is that some of these patients had GCS ≤ 8, indicating deep unconsciousness, which might have led to intubation without additional sedation or analgesia. However, given the limitations in TraumaRegister DGU data entry, analgesia could also be administered but not adequately recorded.

Germany, Austria, and Switzerland have structured emergency medical systems. However, according to the authors’ information, no standardized SOPs exist for prehospital analgesia. Consequently, it is not possible to define exactly which medications were administered. Typically, opioids, ketamine, and NSAIDs are used for prehospital analgesia in these countries, depending on the clinical scenario.

However, the TraumaRegister DGU^®^ currently does not record details of the specific analgesics used, limiting the ability to evaluate adherence to guidelines. Future registry updates should include drug-specific data to enhance the accuracy of prehospital pain management assessments.

Additionally, the proportion of patients receiving analgesia appears to be low due to a misunderstanding of the variable as sedation or anesthesia instead of analgesia during data entry.

Based on the limited assessability of the underlying data, there is a difference in the frequency of analgesia performed in the countries included. While analgesia is performed in 65% in Germany, it is performed in 78% in Austria and even 70% in Switzerland. All three countries use paramedics with different qualifications as a basis for emergency rescue (training in Austria: approx. 1000 h, training in Germany: 4600 h, Switzerland: 5640 h). In contrast, the qualifications of emergency physicians are similar in all countries. In this analysis, 70.6% of patients treated by emergency physicians received analgesia, compared with only 20.4% of those treated by paramedics. A match-pair analysis from the TraumaRegister DGU^®^ a few years ago had shown a significant, but not as large, difference of 69.2% in the physician-treated group versus 53.3% in the paramedic-treated group (*p* < 0.001) [22].

Nevertheless, the underlying rescue service structures are heterogeneous, even within the respective countries. For example, Swiss paramedic training has always included analgesia and is described as being quite successful at 77%, with a comparable 76% (*p* = 0.82) by Swiss emergency physicians, who, however, achieve more sufficient pain reduction in seriously ill/injured patients [23].

For Germany, no holistic performance data is available in emergency medical services, but the heterogeneous approval of medication measures among paramedics is still known [24]. While successful analgesia (pain on hospital transfer NRS < 5 or pain reduction > 2 NRS points) among emergency physicians is 93.3%, for example, it was 36.8% among paramedics in the same area two years ago, with the latest extrapolations showing successful paramedic analgesia of 47% with a range of 22.6–80.6% [21]. Individual studies show very effective and safe paramedic analgesia for both delegated and telemedicine-assisted analgesia [25–30].

However, local guidelines, legal frameworks, and differences in medication availability could also contribute to these variations. Further research is needed to determine whether these differences impact country-specific EMS protocols or transport methods (e.g., higher use of HEMS in Austria/Switzerland). In order to ensure optimal trauma pain management, future efforts should focus on standardizing prehospital analgesia guidelines across different healthcare systems and improving data collection in the TraumaRegister DGU. Furthermore, enhanced research and clinical decision-making would be achieved by improving data collection in the TraumaRegister DGU by distinguishing analgesia from sedation and recording specific drug administration details.

Of those transported by land, 60% (*n* = 43,163) received analgesia before hospitalization, compared with 82% of patients transported via HEMS. Generally, the quality requirements for working in HEMS are higher (e.g., medical specialist, various additional certificates, intensive care experience). This could be one reason for the increased incidence of analgesia in this group. However, this discrepancy inevitably raises the question of whether this difference in care provided by the medical profession is acceptable from the patient’s point of view, as paramedics already provide better analgesia in an individual comparison using Switzerland as an example [23].

On average, patients in the analgesic group spent around 7 min longer on scene than patients who did not receive analgesics. The reasons for this are most likely multifactorial. However, this correlation could be due to the greater severity of injuries in patients in the analgesic group. This hypothesis could also be supported by the fact that 95% of patients in the analgesic group received concomitant fluid therapy. An analysis from the TraumaRegister DGU^®^ also showed in a multivariate method that the on-scene time is extended by an average of 3.7 min with analgesia and by 3.8 min with infusion therapy [31]. The average number of prehospital measures was 2.5 ± 0.89 in the analgesia group, compared to 0.90 ± 0.71 in the non-analgesia group, indicating an increased time expenditure in our present evaluation.

To ensure safe analgesia, it is essential to have a thorough understanding of the pharmacological properties of the substances involved, receive proper training in their use, and have access to emergency equipment to address any complications [5, 26, 32]. This applies to all providers, whether nurses, paramedics, or emergency physicians, and in all situations, whether prehospital or hospital. The necessary monitoring measures and emergency equipment depend on the anticipated complications and side effects. Monitoring for analgesia can be based on the expected side effects. However, unexpected changes in vital signs must be expected in critical trauma care, regardless of analgesia, so monitoring their ECG, blood pressure, respiratory rate, heart rate, and SpO_2_ is essential. Continuous capnography monitoring should be mandatory for all intubated patients. Nasal capnography can also be used in spontaneously breathing patients [32, 33]. Addressing potential insufficiency of analgesia requires education and training, understanding of patients personal and cultural expressions of pain and appropriate documentation as required by the guidelines. Physicians, paramedics and qualified nursing staff need to reflect on the actions of the emergency services to achieve this goal.

### Limitation

The main limitation is the lack of specificity in defining the variable ‘analgosedation’, which could explain some distortions. Additionally, due to the lack of information about the medical staff (such as age, gender, profession, specialty, etc.) and the patient’s pain details, only one-sided analyses can be presented. Ideally, the documentation should be optimized comprehensively, especially since the current guideline has included analgesia as a separate chapter. Digital documentation of emergency medical services could offer additional possibilities for data linking and improved data validity with good software, even if the entry in the TraumaRegister DGU^®^ currently involves processes. The retrospective nature of our study is also one of the limitation. However, it is important to note that the AIS score estimates the severity of the injury rather than the impact of energy input. Additionally, the accident mechanism can only be differentiated rudimentarily due to the lack of detailed information recorded in the TR-DGU.

## Conclusions

Analgesia represents a crucial aspect of emergency medicine, as evidenced by its inclusion in the relevant guidelines. Consequently, it is imperative to ensure that analgesia is documented with precision. However, the present analysis is limited in its ability to assess analgesia, and the reasons for influencing factors cannot always be elucidated. Nevertheless, there appears to be an insufficiency of analgesia in some areas of trauma care. Although the data should be interpreted with caution, our analysis revealed that 34% of patients with a mean Injury Severity Score (ISS) of 19.59 had not received prehospital analgesia. The integration of digital linking between prehospital documentation and clinical data could potentially enhance the quality of the latter.

## Data Availability

Data are provided by the TraumaRegister DGU^®^. Data are available from the TraumaRegister DGU^®^ for researchers who meet the criteria for access to confidential data.

## Abbreviations

AIS: Abbreviated Injury Score
CPR: Cardiopulmonary Resuscitation
DGU: German Trauma Society
ECG: Electrocardiogramm
GCS: Glasgow Coma Scale
HEMS: Helicopter Emergency Medical Services
ICU: Intensive Care Unit
ISS: Injury Severity Score
ITLS: International Trauma Life Support
NRS: Numeric Rating Scale
PHTLS: Prehospital Trauma Life Support
SD: Standard Deviation
SpO2: Saturation of Peripheral Oxygen
SOP: Standard Operating Procedure

## References

[1] Jennings PA, Cameron P, Bernard S. Epidemiology of prehospital pain: an opportunity for improvement. Emerg Med J. 2011;28(6):530–1. 20679429 10.1136/emj.2010.098954

[2] Debus F, et al. Numbers of severely injured patients in Germany. A retrospective analysis from the DGU (German Society for Trauma Surgery) Trauma Registry. Dtsch Arztebl Int. 2015;112(49):823–9. 26754119 10.3238/arztebl.2015.0823 PMC4711294

[3] Matthes G, et al. [Essential measures for prehospital treatment of severely injured patients: the trauma care bundle]. Unfallchirurg. 2015;118(8):652–6. 26160129 10.1007/s00113-015-0042-7

[4] Stork B. K Hofmann-Kiefer 2009 Analgesia as an important component of emergency care. Anaesthesist 58 6 639–48 quiz 649– 50. 19562402 10.1007/s00101-009-1585-1

[5] Kumle B, et al. [Pain therapy in emergency medicine. Focus on emergency admissions]. Anaesthesist. 2013;62(11):902–8. 24173544 10.1007/s00101-013-2247-x

[6] Albrecht E, et al. Undertreatment of acute pain (oligoanalgesia) and medical practice variation in prehospital analgesia of adult trauma patients: a 10 year retrospective study. Br J Anaesth. 2013;110(1):96–106. 23059961 10.1093/bja/aes355

[7] Thomas SH, Shewakramani S. Prehospital trauma analgesia. J Emerg Med. 2008;35(1):47–57. 17997072 10.1016/j.jemermed.2007.05.041

[8] Carr DB, Goudas LC. Acute pain. Lancet. 1999;353(9169):2051–8. 10376632 10.1016/S0140-6736(99)03313-9

[9] Hossfeld B, et al. [Prehospitale analgesia in adults]. Anasthesiol Intensivmed Notfallmed Schmerzther. 2016;51(2):84–95. quiz 96. 26949902 10.1055/s-0042-101466

[10] Oberholzer N, et al. Factors influencing quality of Pain Management in a physician staffed Helicopter Emergency Medical Service. Anesth Analg. 2017;125(1):200–9. 28489643 10.1213/ANE.0000000000002016

[11] Tait RC, Chibnall JT, Kalauokalani D. Provider judgments of patients in pain: seeking symptom certainty. Pain Med. 2009;10(1):11–34. 18992039 10.1111/j.1526-4637.2008.00527.x

[12] Weisse CS, Sorum PC, Dominguez RE. The influence of gender and race on physicians’ pain management decisions. J Pain. 2003;4(9):505–10. 14636818 10.1016/j.jpain.2003.08.002

[13] Brokmann JC, et al. Analgesia by telemedically supported paramedics compared with physician-administered analgesia: a prospective, interventional, multicentre trial. Eur J Pain. 2016;20(7):1176–84. 26914284 10.1002/ejp.843

[14] Brennan F, Carr DB, Cousins M. Pain management: a fundamental human right. Anesth Analg. 2007;105(1):205–21. 17578977 10.1213/01.ane.0000268145.52345.55

[15] Helm M, et al. Oligoanalgesia in patients with an initial Glasgow coma scale score ≥ 8 in a physician-staffed Helicopter Emergency Medical Service: a multicentric secondary data analysis of > 100,000 out-of-hospital emergency missions. Anesth Analg. 2020;130(1):176–86. 31335406 10.1213/ANE.0000000000004334

[16] Chambers JA, Guly HR. The need for better prehospital analgesia. Arch Emerg Med. 1993;10(3):187–92. 8216592 10.1136/emj.10.3.187 PMC1285986

[17] Häske D, et al. Comparison of manual statements from out-of-hospital trauma training programs and a national guideline on treatment of patients with severe and multiple injuries. Eur J Trauma Emerg Surg. 2022;48(3):2207–17. 34426883 10.1007/s00068-021-01768-z

[18] Deutsche Gesellschaft für Unfallchirurgie e.V. S3-Leitlinie Polytrauma/Schwerverletzten-Behandlung (AWMF Registernummer 187– 023), V., verfügbar unter https://www.awmf.org/leitlinien/detail/ll/187-023.html. Zugriff am 22.01.2024., S3-Leitlinie Polytrauma/Schwerverletzten-Behandlung. 2022.

[19] e.V., D.G.f.U., S3-Leitlinie Polytrauma/Schwerverletzten-Behandlung. (AWMF Registernummer 187– 023), Version 4.0. 2022.

[20] Eimer C, et al. Prehospital analgesia in pediatric trauma and critically ill patients: an analysis of a German air rescue service. Scand J Trauma Resusc Emerg Med. 2023;31(1):5. 36709289 10.1186/s13049-023-01069-x PMC9883913

[21] Jahresbericht S-B. SQR-BW (2023) Jahresbericht 2022 Rettungsdienst Baden-Württemberg. 2022.

[22] Bieler D et al. Does the presence of an emergency physician influence prehospital time, prehospital interventions and the mortality of severely injured patients? A matched-pair analysis based on the trauma registry of the German Trauma Society (TraumaRegister DGU(^®^)). Injury, 2017. 48(1): pp. 32–40. 10.1016/j.injury.2016.08.015 27586065

[23] Kiavialaitis GE, et al. Clinical practice of prehospital analgesia: an observational study of 20,978 missions in Switzerland. Am J Emerg Med. 2020;38(11):2318–23. 31785972 10.1016/j.ajem.2019.10.033

[24] Vilcane S, Scharonow O, Weilbach C, Scharonow M. Application of analgesics in emergency services in Germany: a survey of the medical directors. BMC Emerg Med. 2023;23(1):104. 37710177 10.1186/s12873-023-00878-8 PMC10500886

[25] Häske D, Schempf B, Gaier G, Niederberger C. [Prehospital analgesia performed by paramedics: quality in processes and effects under medical supervision]. Anaesthesist. 2014;63(3):209–16. 24562597 10.1007/s00101-014-2301-3

[26] Häske D, et al. Analgesia in patients with trauma in Emergency Medicine. Dtsch Arztebl Int. 2017;114(46):785–92. 29229039 10.3238/arztebl.2017.0785 PMC5730701

[27] Häske D, et al. Efficacy and safety in ketamine-guided prehospital analgesia for abdominal pain. Intern Emerg Med. 2022;17(8):2291–7. 36205836 10.1007/s11739-022-03091-w

[28] Kill C, Wranze GI, Hartmann E, Hündorf H, Gliwitzky HP, Wulf B. H Kompetenzentwicklung Im Rettungsdienst Notfall Rettungsmed. 2007;10(4):266–72.

[29] Scharonow M, Alberding T, Oltmanns W, Weilbach C. Project for the introduction of prehospital analgesia with fentanyl and morphine administered by specially trained paramedics in a rural service area in Germany. J Pain Res. 2017;10:2595–9. 29158691 10.2147/JPR.S151077 PMC5683795

[30] Gnirke A, et al. [Analgesia in the emergency medical service: comparison between tele-emergency physician and call back procedure with respect to application safety, effectiveness and tolerance]. Anaesthesist. 2019;68(10):665–75. 31489458 10.1007/s00101-019-00661-0

[31] Wyen H, et al. The golden hour of shock - how time is running out: prehospital time intervals in Germany–a multivariate analysis of 15, 103 patients from the TraumaRegister DGU(R). Emerg Med J. 2013;30(12):1048–55. 23258373 10.1136/emermed-2012-201962

[32] Godwin SA, et al. Clinical policy: procedural sedation and analgesia in the emergency department. Ann Emerg Med. 2005;45(2):177–96. 15671976 10.1016/j.annemergmed.2004.11.002

[33] Dewdney C, et al. Capnography for procedural sedation in the ED: a systematic review. Emerg Med J. 2017;34(7):476–84. 27565194 10.1136/emermed-2015-204944

